# Extracting Information Automatically From Biological Literature

**DOI:** 10.1002/cfg.102

**Published:** 2001-10

**Authors:** Christian Blaschke, Robert Hoffmann, Juan Carlos Oliveros, Alfonso Valencia

**Affiliations:** Protein Design Group National Center for Biotechnology CNB-CSIC Cantoblanco Madrid E-28049 Spain


**Comparative and Functional Genomics**
*Comp Funct Genom* 2001; **2**: 310–313.
**DOI:** 10.1002/cfg.102

**Conference Review**

### Extracting information automatically from biological literature

### A presentation for the ESF workshop 'Proteomics: Focus on Protein Interactions'

Christian Blaschke, Robert Hoffmann, Juan Carlos Oliveros and Alfonso Valencia*
*Protein Design Group, CNB-CSIC, Madrid E-28049, Spain*

**Correspondence to:*
*A. Valencia, Protein Design Group, National Center for Biotechnology, CNB-CSIC, Cantoblanco, Madrid E-28049, Spain.*
*E-mail: valencia@cnb.uam.es*

Received: 4 July 2001
Accepted: 27 July 2001

**Keywords: information in text sources; genomics; proteomics**

### Information contained in sources of biological text

In the past few decades, biologists have generated a large amount of data that has been published mainly in biological journals. It is now important to be able to recover as much as possible of this information as it constitutes a precious source of additional information for helping to understand the new genomics and proteomics data. More than 10 million abstracts of such papers are contained in the Medline collection and are available on the World Wide Web Via PubMed [10], and this collection will expand considerably once journals become freely available on the Web (PubMed Central [15], E-BioSci [7]).

In parallel with these plain text information sources, basic molecular biology data has been stored in various semi-structured repositories, such as protein and gene sequence databases, and more recently in databases of protein structures, protein interactions, transcription factors, point mutations, metabolic pathways and many others.

There is a commonly-recognized need for linking and complementing the information contained in these databases with the information stored in the literature, a task that right now requires detailed work by scientists and in some cases database users.

### Ways of extracting information automatically from text

Three main types of systems are being developed:

- *Statistical methods*. These are based on the frequency of occurrence of words in a large text corpus that has been previously organized in line with some form of external knowledge (for example, groups of genes with similar expression patterns or proteins that belong to the same protein family). Significant patterns detected, and the information associated with them, are used to characterize the corresponding groups of genes or proteins.
- *Computational linguistics methods*. These methods use parsers and grammars to extract syntactic information and internal dependencies within individual sentences. This approach is quite general and can be applied to different

Abbreviations: Information extraction in Biology

Copyright © 2001 John Wiley & Sons, Ltd.

knowledge domains after careful adaptation to the specific problems of the field. It is important to realize that there is still no guarantee that this adaptation can be successfully achieved for the field of molecular biology.

- *Frame-based approaches*. A third type of approach combines features of the two previous methods with a set of previously defined templates for possible textual relationships, called frames. In common with computational linguistics methods, this approach can make use of syntactic information although it can also work without prior parsing and tagging of the text. As in the case of statistical methods, it uses scoring schemes that depend on the number of occurrences of particles in large collections of text.

### Overview of publications on automated information extraction as applied to molecular biology

### Statistical approaches

- Extraction of keywords from Medline abstracts in order to qualify the function of previously classified protein families [2].
- Assistance in the annotation of experimental results obtained from DNA expression arrays [4].
- Distribution of extracted terms in order to classify them in relation to articles linked to the OMIM database of human disease [1].
- Statistical and linguistic techniques for building knowledge bases and domain-specific thesauri [11].
- Classification of protein sub-cellular localization [6].

### Detection of protein names in biomedical texts

- Combination of syntactic information and morphological differences in comparison with the surrounding text [8,13].

### Protein-protein interaction detection systems

- Co-occurrence of protein names within the same abstract, and whether this implies a functional relationship or not [19,9].
- Use of frame-based methods to extract large sets of protein-protein interactions [3].
- Evaluation of the degree of relationship between experimental protein interactions (DIP database, [21]) and underlying text sources [5].
- Restricted application of linguistic methods and various grammars to reduced systems in order to demonstrate potential applicability [14,16,17,18,20,12].

### Commercial tools and systems

A number of companies have announced the commercialization of basic tools such as part-of-speech taggers and parsers, databases with manually-organized sets of text as well as various information extraction systems for the detection and classification of information. Among others, there are general purpose tools from IBM and Xerox, and more specialized systems from companies such as Autonomy, SAS, Ingenuity, Semio, SRA and Temis.

### Two systems for automated information extraction

We describe here two previously published systems for which detailed evaluation of the results is available. The first represents a typical statistical approach, based on the extraction of keywords from pre-organized text groups, while the second is an example of a hybrid approach based on a set of pre-defined frames related to protein interactions.

### Geisha

Geisha (Gene Expression Information System for Human Analysis [4]) is conceptually similar to other statistical approaches, such as that previously developed by Andrade and Valencia [2] for the assignment of functional keywords to protein families. The Geisha system involves the annotation of function for groups of genes that show similar expression patterns in DNA array experiments. First the system uses the groups of genes as a framework for clustering the related literature. In a second step it estimates the frequency of relevant words in the various literature clusters, and then in a third step these frequencies are compared in order to assess their statistical relevance (in the form of Z-scores). A similar procedure is applied to the extraction of complete sentences specific to the various gene clusters.

Since biological information is often expressed in composite terms such as ‘DNA polymerase’ and

Copyright © 2001 John Wiley & Sons, Ltd. 

'RNA polymerase', these constructions are detected by analyzing the frequency of these co-occurrences in comparison to the expected frequency of the individual component words.

The results of the Geisha system have been extensively compared to the annotations provided by databases and human experts, showing how in many cases Geisha was able to extract relevant or alternative information to that provided by other sources.

### Suiseki

Suiseki was designed as an integrated system for the extraction of protein interactions from Medline abstracts [5]. The system includes some features of computational linguistics methods (such as text tagging) and some from statistical methods (such as the use of statistics relating to word occurrence), together with a collection of pre-established frames that capture possible ways of expressing interactions in biological text.

The steps followed by Suiseki are: (a) download of Medline abstracts (or local access); (b) part-of-speech tagging for the detection of protein names; (c) detection of protein name synonyms; (d) determination of verbs indicating a relationship (interaction keywords); (e) extraction of protein-protein interactions with a minimal set of nineteen frames; (f) exclusion of negative interactions with a set of specific frames; (g) use of the frame scores (proportional to their accuracy in describing interactions), and the number of sentences matching each frame, to obtain scores for each interaction; (h) finally, the interactions are stored in a database of relationships and represented via a dynamic web interface that allows simultaneous manipulation of the underlying information (names and interactions), access to the text sources (sentences corresponding to the interactions and Medline data) and manipulation by human experts.

The frames used by the Suiseki system include general patterns such as 'protein A -particle indicating interaction- protein B', where various particles can indicate interaction (e.g. bind, phosphorylates), as well as more specialized patterns (e.g. 'phosphorylation/binding/ ... of protein A ... by protein B'; 'complex of protein A and protein B') that can be more accurate than general patterns, at the expense of covering a smaller number of cases.

The Suiseki system requires minimal user intervention, and can easily be applied to large collections of written text.

To evaluate their systems, various authors have employed sets of sentences that have previously been analyzed manually. More recently, it has become clear that evaluating the number of known interactions that can be retrieved automatically could provide more biologically realistic information. In particular, the detection of gene and protein names remains as the main problem in this field, and evaluation using a set of sentences tends to avoid this problem by simply assuming that the names are already known. In contrast, evaluations carried out using large collections of known interactions directly involve the problem of relating those names used in databases and those used in the literature. For example, in the case of the interactions held in the DIP database, more than 40% of the entries contain names that could not be found in any Medline entry [5], and this gives some indication of the severity of the protein name problem.

In the case of the Suiseki system, for random sets of sentences the correct interactions were extracted in 30% of cases, but more importantly, for the most frequent interactions as many as 80% of the interactions extracted were correct.

### References

1. Andrade MA, Bork P. 2001. Automatic extraction of information in molecular biology. *FEBS Lett* **476**: 12–17.
2. Andrade MA, Valencia A. 1998. Automatic extraction of keywords from scientific text: Application to the knowledge domain of protein families. *Bioinformatics* **14**: 600–607.
3. Blaschke C, Andrade MA, Ouzounis C, Valencia A. 1999. Automatic extraction of biological information from scientific text: protein-protein interactions. *Proc Int Conf Intell Syst Mol Biol* **1999**: 60–67.
4. Blaschke C, Oliveros JC, Valencia A. 2001. Mining functional information associated with expression arrays. *Funct Integrative Genom* **1**(4): 256–268.
5. Blaschke C, Valencia A. 2001. Can bibliographic pointers for known biological data be found automatically? Protein interactions as a case study. *Comp Funct Genom* **2**(4): 196–206.
6. Craven M, Kumlien J. 1999. Construction of biological knowledge bases by extracting information from text sources. *Proc Int Conf Intell Syst Mol Biol* **1999**: 77–86.
7. E-BioSci 2001. The Electronic Publication Initiative at EMBO: **http://www.embo.org/E_Pub_pages.html**
8. Fukuda K, Tsunoda T, Tamura A, Takagi T. 1998. Information extraction: identifying protein names from biological papers. *Pac Symp Biocomput* **1998**: 707–718.
9. Jenssen TK, Lægreid A, Komorowski J, Hovig E. 2001. A literature network of human genes for high-throughput analysis of gene expression. *Nat Genet* **28**: 21–28.

Copyright © 2001 John Wiley & Sons, Ltd. 

10. Medline 2001. PubMed database at the National Library of Medicine: **http://www.ncbi.nlm.nih.gov/entrez/query.fcgi**
11. Ohta Y, Yamamoto Y, Okazaki T, Uchiyama I, Takagi T. 1997. Automatic construction of knowledge base from biological papers. *Proc Int Conf Intell Syst Mol Biol* **1997**: 218–225.
12. Park JC, Kim HS, Kim JJ. 2001. Bidirectional incremental parsing for automatic pathway identification with combinatory categorial grammar. *Pac Symp Biocomput* **2001**: 396–407.
13. Proux D, Rechenmann F, Julliard L, Pillet V, Jacq B. 1998. Detecting gene symbols and names in biological texts: a first step toward pertinent information extraction. *Genome Inform Ser Workshop Genome Inform (* **GIW98**): 72–80.
14. Proux D, Rechenmann F, Julliard L. 2000. A pragmatic information extraction strategy for gathering data on genetic interactions. *Proc Int Conf Intell Syst Mol Biol* **2000**: 279–285.
15. PubMed Central 2001. A digital archive of life sciences journal literature managed by the National Center for Biotechnology Information (NCBI): **http://www.pubmedcentral.nih.gov/**
16. Rindflesch TC, Hunter L, Aronson AR. 1999. Mining molecular binding terminology from biomedical text. *Proc AMIA Symp* **1999**: 127–131.
17. Rindflesch TC, Tanabe L, Weinstein JN, Hunter L. 2000. EDGAR: Extraction of drugs, genes and relations from the biomedical literature. *Pac Symp Biocomput* **2000**: 515–524.
18. Sekimizu T, Park HS, Tsujii J. 1998. Identifying the interaction between genes and gene products based on frequently seen verbs in Medline abstracts. *Genome Inform Ser Workshop Genome Inform (* **GIW98**): 62–71.
19. Stapley BJ, Benoit G. 2000. Biobibliometrics: information retrieval and visualization from co-occurrences of gene names in Medline abstracts. *Pac Symp Biocomput* **2000**: 529–540.
20. Thomas J, Milward D, Ouzounis C, Pulman S, Carrol M. 2000. Automatic extraction of protein interactions from scientific abstracts. *Pac Symp Biocomput* **2000**: 538–549.
21. Xenarios I, Rice DW, Salwinski L, Baron MK, Marcotte EM, Eisenberg D. 2000. DIP: the Database of Interacting Proteins. *Nucleic Acids Res.* **28**: 289–291.

Copyright © 2001 John Wiley & Sons, Ltd. 
